# Supplementary figures and images for: The Structural Determinants of Intra-Protein Compensatory Substitutions
Source: Mol Biol Evol. 2022 Mar 29;39(4):msac063. doi: 10.1093/molbev/msac063 (PMC9004419; doi:10.1093/molbev/msac063)

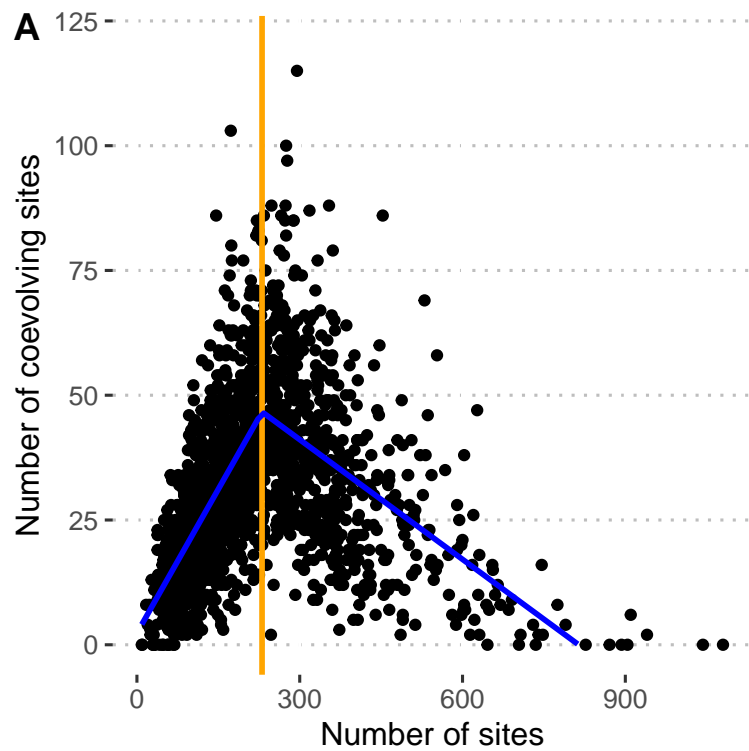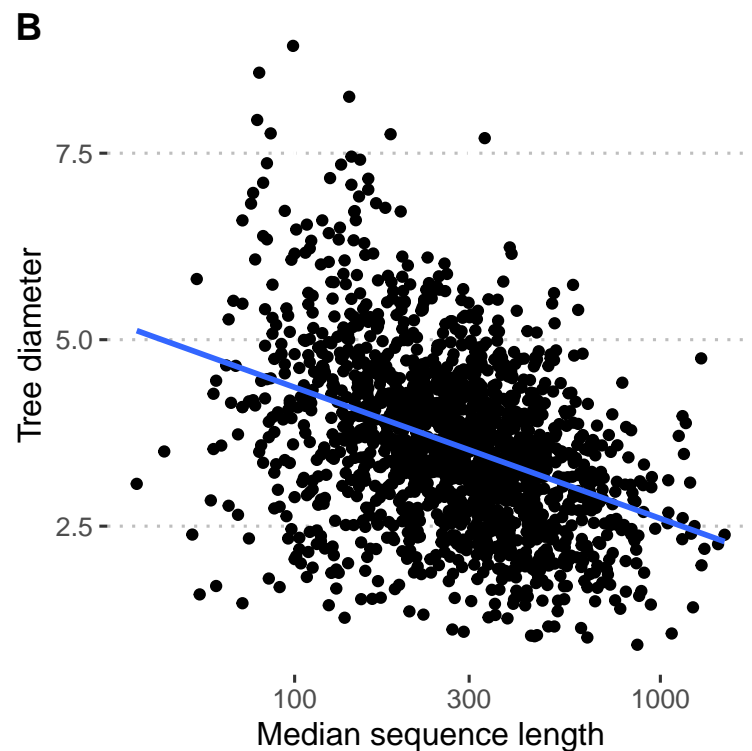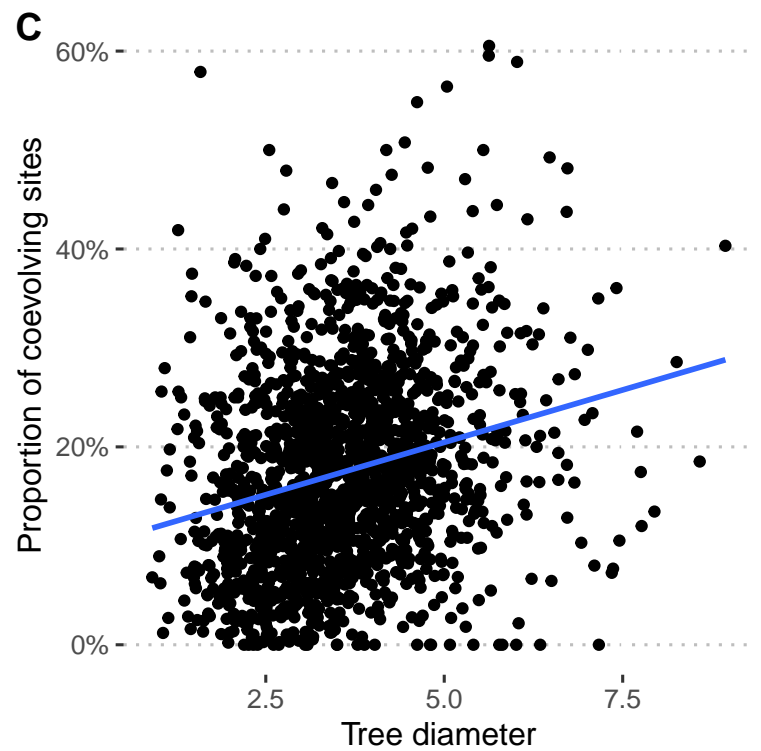

Supplement: msac063_Supplementary_Data [file msac063_supplementary_data.zip › FigureS1.pdf]

Standardized rate

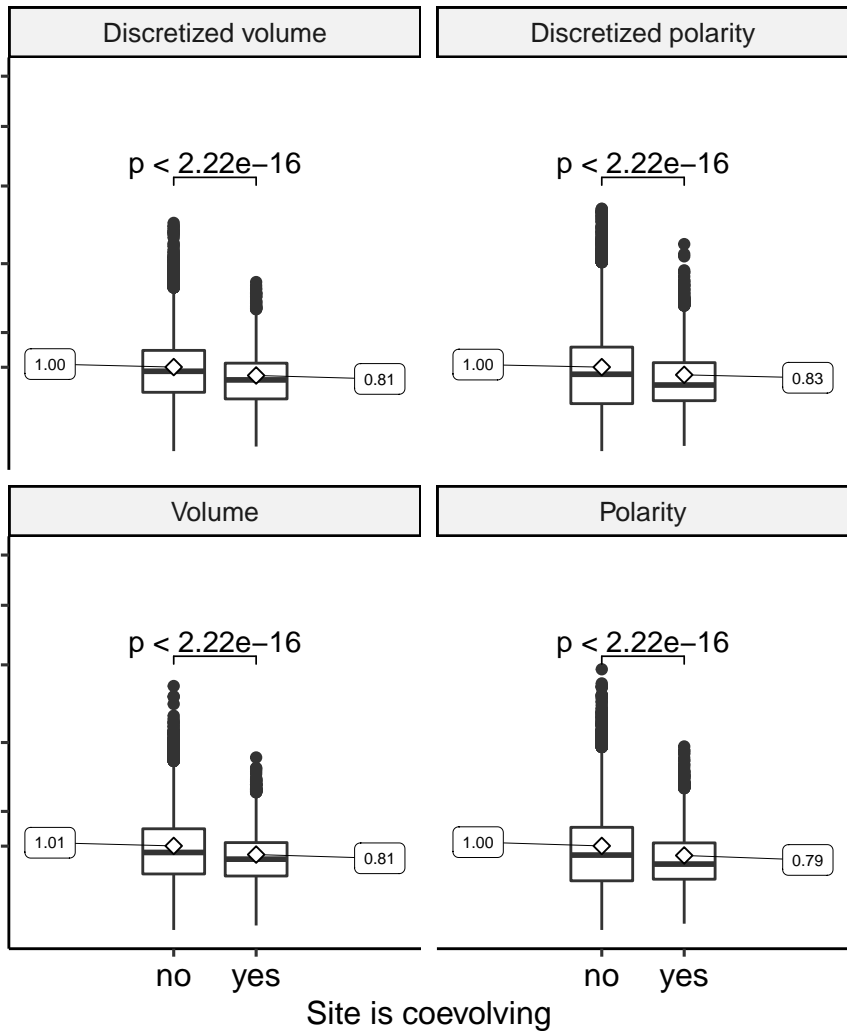

Supplement: msac063_Supplementary_Data [file msac063_supplementary_data.zip › FigureS2.pdf]

**A**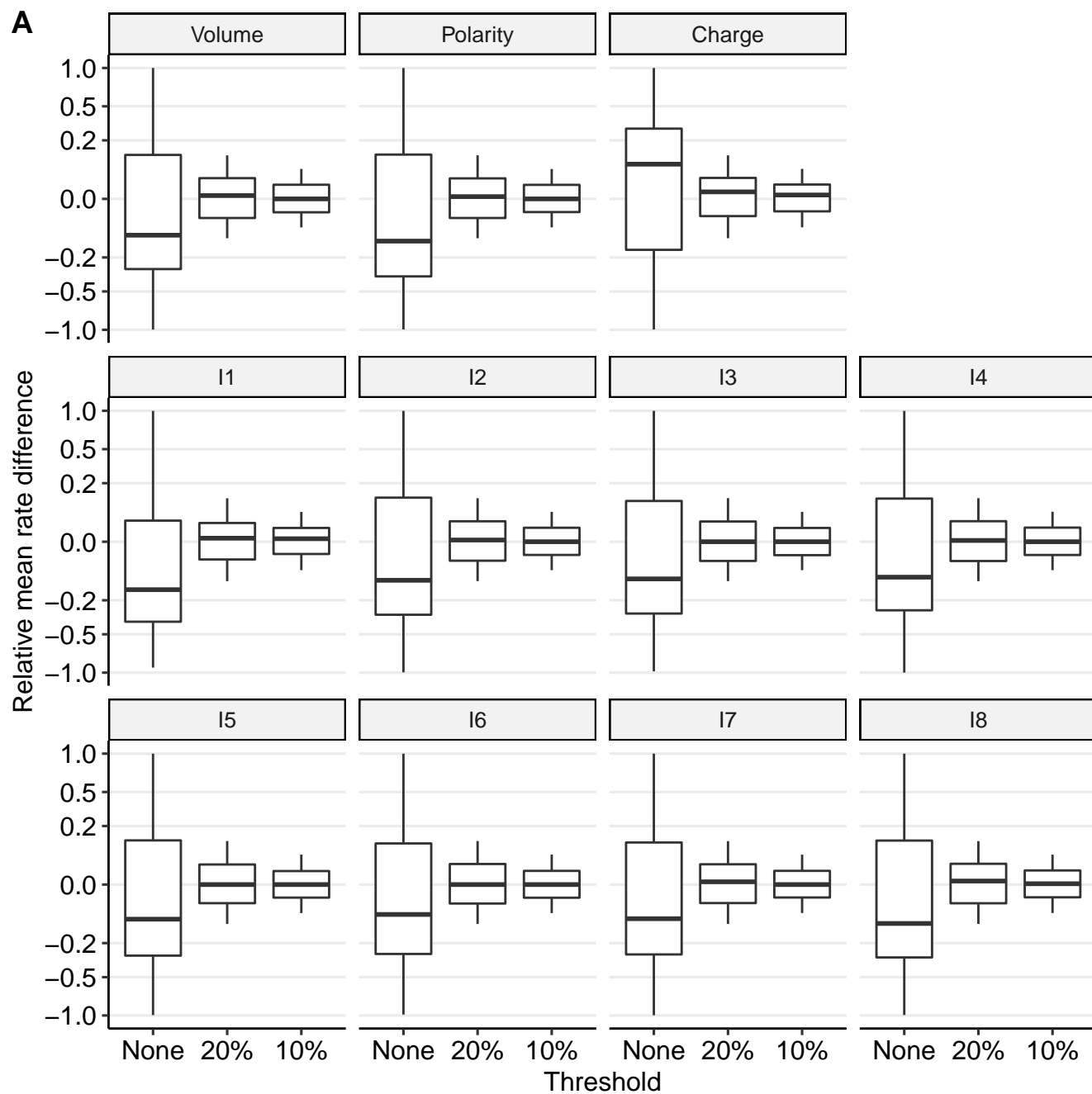**B**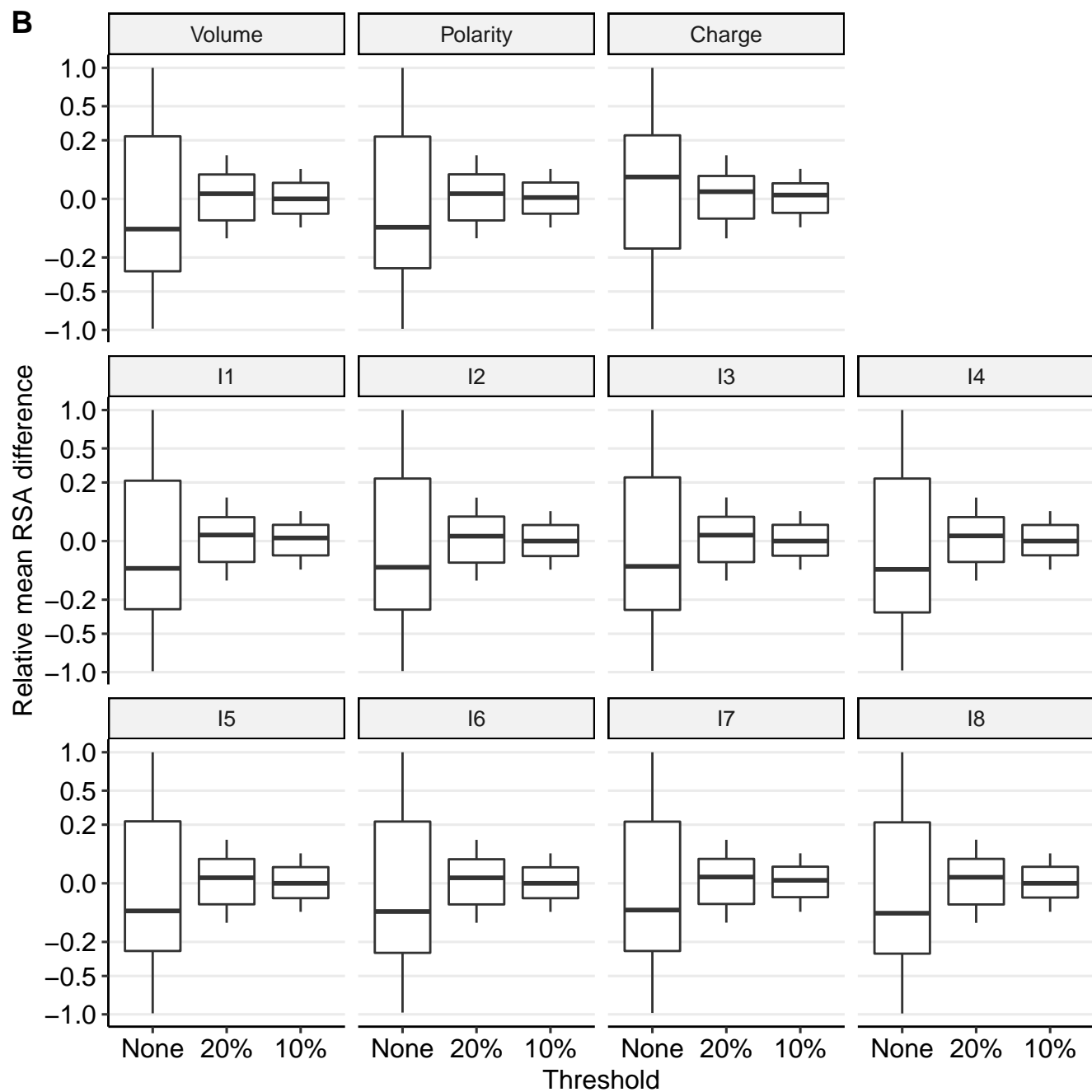

Supplement: msac063_Supplementary_Data [file msac063_supplementary_data.zip › FigureS4.pdf]
