# Supplementary material for: The Structural Determinants of Intra-Protein Compensatory Substitutions
Source: Mol Biol Evol. 2022 Mar 29;39(4):msac063. doi: 10.1093/molbev/msac063 (PMC9004419; doi:10.1093/molbev/msac063)
Supplement: msac063_Supplementary_Data [file FigureS3.pdf › FigureS3.pdf]

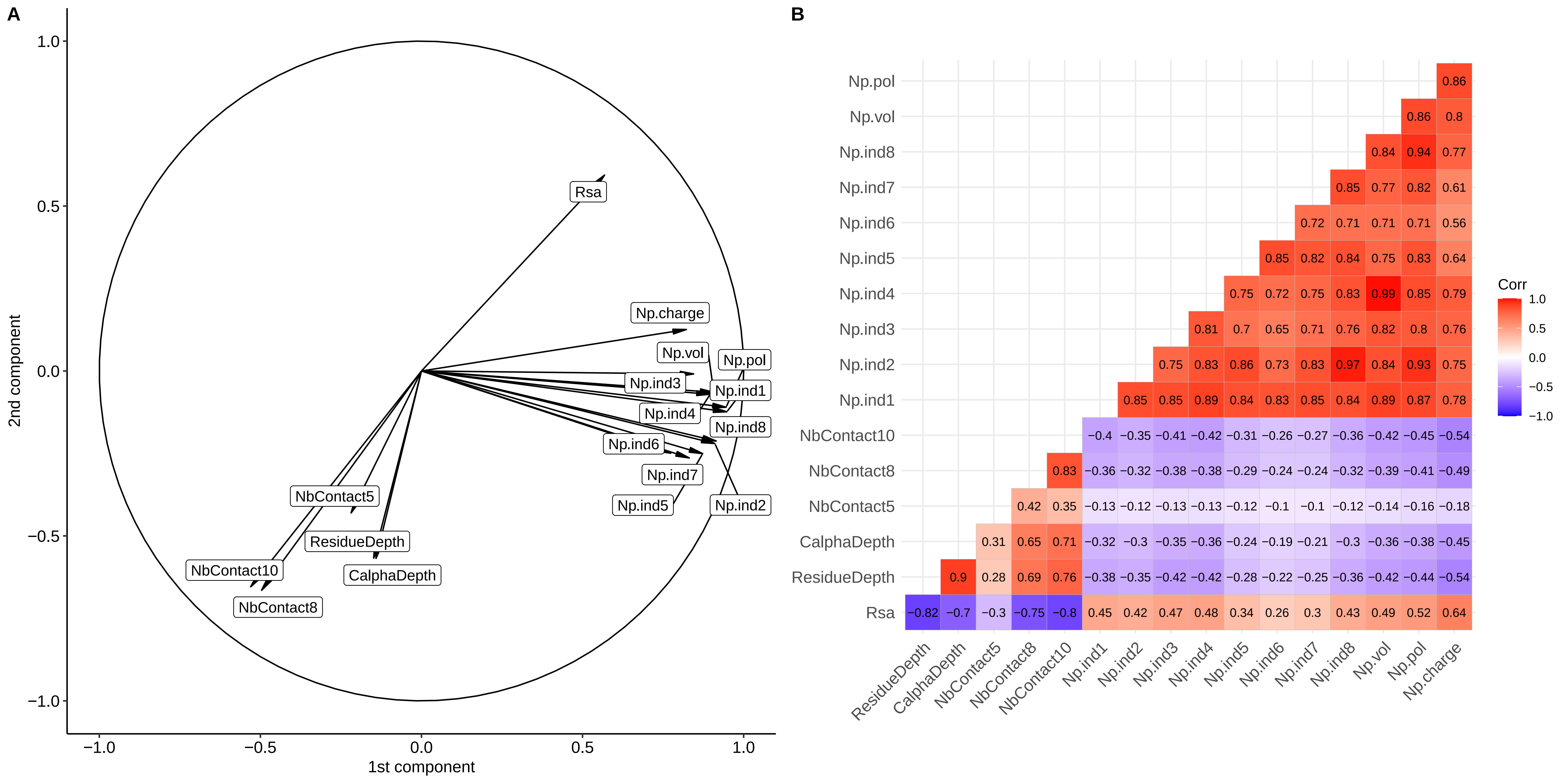
A
1.0
0.5
0.0
−0.5
−1.0
2nd component
−1.0
−0.5
0.0
0.5
1.0
1st component
Rsa
Np.charge
Np.vol
Np.pol
Np.ind3
Np.ind1
Np.ind4
Np.ind8
Np.ind6
Np.ind7
Np.ind5
Np.ind2
NbContact5
ResidueDepth
NbContact10
CalphaDepth
NbContact8
B
Np.pol 0.86
Np.vol 0.86 0.8
Np.ind8 0.84 0.94 0.77
Np.ind7 0.85 0.77 0.82 0.61
Np.ind6 0.72 0.71 0.71 0.71 0.56
Np.ind5 0.85 0.82 0.84 0.75 0.83 0.64
Np.ind4 0.75 0.72 0.75 0.83 0.99 0.85 0.79
Np.ind3 0.81 0.7 0.65 0.71 0.76 0.82 0.8 0.76
Np.ind2 0.75 0.83 0.86 0.73 0.83 0.97 0.84 0.93 0.75
Np.ind1 0.85 0.85 0.89 0.84 0.83 0.85 0.84 0.89 0.87 0.78
NbContact10 −0.4 −0.35 −0.41 −0.42 −0.31 −0.26 −0.27 −0.36 −0.42 −0.45 −0.54
NbContact8 0.83 −0.36 −0.32 −0.38 −0.38 −0.29 −0.24 −0.24 −0.32 −0.39 −0.41 −0.49
NbContact5 0.42 0.35 −0.13 −0.12 −0.13 −0.13 −0.12 −0.1 −0.1 −0.12 −0.14 −0.16 −0.18
CalphaDepth 0.31 0.65 0.71 −0.32 −0.3 −0.35 −0.36 −0.24 −0.19 −0.21 −0.3 −0.36 −0.38 −0.45
ResidueDepth 0.9 0.28 0.69 0.76 −0.38 −0.35 −0.42 −0.42 −0.28 −0.22 −0.25 −0.36 −0.42 −0.44 −0.54
Rsa −0.82 −0.7 −0.3 −0.75 −0.8 0.45 0.42 0.47 0.48 0.34 0.26 0.3 0.43 0.49 0.52 0.64
ResidueDepth
CalphaDepth
NbContact5
NbContact8
NbContact10
Np.ind1
Np.ind2
Np.ind3
Np.ind4
Np.ind5
Np.ind6
Np.ind7
Np.ind8
Np.vol
Np.pol
Np.charge
Corr
1.0
0.5
0.0
−0.5
−1.0
